# Vesical and Urethral Calculi

**Published:** 1888-07

**Authors:** W. B. Rogers

**Affiliations:** Professor of Principles and Practice of Surgery and Clinical Surgery.—Formerly Professor of Anatomy, Clinical and Genito-Urinary Surgery, in the Memphis Hospital Medical College


DANIEL'S

### TEXAS MEDICAL JOURNAL.

A Representative Organ of the Medical Profession, and an Exponent of Rational Medicine; devoted to the Organization, Advancement and Elevation of the Profession in Texas.

PUBLISHED MONTHLY.—SUBSCRIPTION $2.00 A YEAR.

Vol. IV. AUSTIN, JULY, 1888. No. 1.

### ORIGINAL ARTICLES.

CONTRIBUTED EXCLUSIVELY TO THIS JOURNAL.

*The Articles in this Department are accepted and published with the understanding that we are not responsible for, nor do we indorse the views and opinions of the writers by so doing.*

### VESICAL AND URETHRAL CALCULI.

**Report of Fifteen Cases.**

*By W. B. Rogers, M. D.,*

*Professor of Principles and Practice of Surgery and Clinical Surgery.—Formerly Professor of Anatomy, Clinical and Genito-Urinary Surgery, in the Memphis Hospital Medical College.*

CASE I.—VESICAL CALCULUS, PHOSPHATIC—WEIGHT 370 GRS.

EDWARD S., male, white, nineteen years; of one of the eastern counties of the State of Mississippi, was referred to me by Dr. Bonner. The patient was a son of a physician, and had, for five years complained of cystitis; but no examination of bladder had been made until he entered my Infirmary in February, 1888. Stone was suspected, and this suspicion was confirmed upon first introduction of searcher. Four days later, I performed the perineal section. On reaching the membranous urethra, which I divided for about ¾ of an inch, I introduced a pair of dressing forceps, and gliding along the groove of the staff, entered the bladder, when I opened the blades gently, stretching the neck of that organ; then, with my finger, still further dilated the neck of the bladder, and passed in a pair of heavy, though not too large stone forceps, seized and crushed the stone, which was soft. The fragments were easily washed out. The patient had no rise of temperature after the operation. Had perfect control of his urine, and did not even soil the sheet with a drop of water.

On the sixth day he was able to be up, and on the twelfth day the wound of the perineal section had healed, and he passed water naturally.

The almost perfect harmlessness of the perineal section presents itself to my mind as the proper operation to perform in all cases where difficulty is anticipated, in performing lithotripsy. With this perineal section, and the neck of the bladder dilated gently with finger, we can, with greater certainty, cleanse the bladder of all fragments of stone, and that too, without any complicated suction apparatus, which is necessary when lithotripsy is performed through the whole length of the urethra.

CASE II—VESICAL CALCULUS, LITHIC ACID—WEIGHT 300 GRS.

Wm. H., five years, white, from Coahoma county, Mississippi, was sent to my private infirmary in the fall of 1886, by Dr. Coleman, of Mississippi.

At the age of 2 years, the patient passed a small calculus, per urethra, but symptoms of cystitis gradually increased up to the time of my seeing him. His urine was rankly ammoniacal, his suffering intense, and his physical condition exceedingly poor.

The sound determined the presence of a calculus, and after a few days preparatory treatment I performed the lateral operation. Finding the stone too large for easy abstraction, I crushed it by means of the lithotrite, and succeeded in clearing the bladder of the fragments. The venous hemorrhage was considerable, and told on the already debilitated condition of the patient. It was checked by the use of hot water douche. Four days later he was clear of fever, and on the eighth days was sitting up.

Several weeks elapsed before the wound healed, and some months before he was strong. One year later, he had perfect control over the passage of urine, and was in fine health.

CASE III.—VESICAL CALCULUS, PHOSPHATIC—WEIGHT 300 GRS.

Louis Vici, age 47 years, a native of Italy, by profession a musician, and at the time of my first consultation, was traveling with the Abbott Opera Troupe. Was admitted to my private infirmary in December, 1887. The history was one of severe cystitis, lasting over a period of seven years. He had been examined and treated in hospitals, both in Brooklyn and New York, for stricture of urethra.

My first examination detected two strictures, the smaller being 10 calibre, (F.) in the bulbous urethra, passing which I entered the bladder, and detected a calculus.

It was with much persuasion that I gained his consent to the operation for stone, on account of his doubts as to the existence of the stone. His urine was putrid, and one-third of what passed from the bladder was blood and mucus.

So tortuous was the canal, and complicated by false passages, that I was unable, after three examinations, to find the route to the bladder. But after suitable preparations, I made perineal section on staff, in one of the false passages, and then succeeded in finding the urethra in the membranous portion, opened it, and following it first forward, divulsed the posterior and incised the anterior stricture, then sought the vesical end, and entered the bladder, and after a prolonged search, failed to find the calculus. I was positive that the stone was there, but desisted, because of the operation having been already necessarily prolonged. His condition was poor. He rallied, and the next day the pain in his bladder was undiminished, although water had free exit. I

passed No. 32 (F) sound into the bladder, (for the purpose of keeping canal patulous,) and sought for the calculus, and again failed to find it. Two days later, the same instrument failed to detect the stone. Fever came on, an abscess in ischio-rectal region developed, and for ten days his condition was almost beyond hope of recovery. In order to give *free* vent to pus, I anæsthetized, and opened *widely* the abscess; and then through the perineal opening passed a sound, which came immediately in contact with the calculus. The next day I invited all who had witnessed the failure to find the stone, to be present. I then enlarged the neck of the bladder, as in the lateral operation, and extracted a stone measuring 4½ inches by 3 inches in long and short circumferences. All pain in bladder ceased, and his recovery was rapid. He left eighteen days later, saying he was a new man. Before leaving, I passed No. 32 sound, found canal patulous; impressed upon him the necessity of regular introduction of sound.

This case illustrates the difficulty sometimes encountered both in detecting a stone before operating, as well as that of finding it even when the finger is passed into the bladder.

CASE IV.—VESICAL CALCULUS, PHOSPHATIC—WEIGHT 380 GRS.

W. T. W., 36 years, male, white, was sent to my private infirmary by Dr. Watson, of Mississippi, in June, 1885, for treatment for stone.

The patient was much emaciated, and a great sufferer with cystitis; urine containing blood, mucus and pus, and his rectum protruded quite three inches during efforts to void urine, and to this organ several physicians had directed treatment. His history showed irritability of the bladder, which had existed several years, but had grown much worse during the past year. It was also known that he had been taught to pass straws into his urethra; and one year ago he told of a stick, introduced into that canal, getting away from him, and passing into the bladder.

The patient was an imbecile, and though not violent ordinarily, it required two assistants to aid me, before I succeeded in anæsthetizing him, and with sound I confirmed the diagnosis of stone. A stricture was also encountered, half an inch within the meatus.

Proper preparatory treatment for a period of ten days, put the patient in a fair condition, when the operation was performed.

Assisted by Drs. Watson and Ashe, I divided the stricture and performed the lateral operation. The stone, upon being grasped with the forceps, was found rather large for easy extraction, and for the purpose of changing its axis, my finger was passed along the incision, when it encountered a piece of wire, projecting into the floor of the wound. Taking a pair of hæmostatic forceps I succeeded in pulling out the wire which lay imbedded in the prostate gland. The stone was then extracted, and a careful examination failed to find any other foreign body or stone. The loss of blood was trifling. There was no rise of temperature after the operation. On the seventh day my patient was up, and on the twelfth day the incision was healed.

The stone was about the size and shape of a pigeon egg. The wire measured about two inches in length but was broken in three pieces during my efforts at extraction. It had evidently been passed into the bladder, and by contraction of this organ had been driven into the prostate gland.

In this instance lithotripsy was rejected because of suspected foreign body as nucleus to stone. The supra-pubic operation was not done because of the difficulty expected in managing patient during after-treatment.

It would almost seem providential that I selected the only route which could have afforded relief. Any other route would have removed the stone but missed the wire, which, if left in the prostate gland, would have kept up the cystitis, and repeated examination would have led to the conclusion of pyelitis.

CASE V.—URETHRAL CALCULUS—URIC ACID.—WEIGHT 165 GRS.

Wm. H—, male; white; six years, from Clarksdale, Miss.; was brought to my infirmary, March 20, '88, in intense pain. Examination showed bladder distended, with urine and calculus in membranous urethra.

I first saw him at midnight and gave opiate; he rested well for six hours, and passed water. He was placed on the table and perineal section made, with calculus as guide. It was found lying in quite a pouch—or dilated portion of the canal—in front of which was a stricture of —— calibre; this was divided through the perineal wound. The bladder was explored and no other stone discovered. He went home the following morning.

This patient was operated on eighteen months before—see No. II.

The patient was perfectly well, up to four days before coming to me, when he was taken with violent pain in perineum and abdomen. The pain was mostly due to distention of bladder, the stone acting as a plug, closing the urethra.

CASE VI.—VESICAL CALCULUS, PHOSPHATIC—WEIGHT 540 GRS.

Susan Page, colored, 34 years of age, city, was the subject of a vesico-vaginal opening, and presented at my college clinic in 1881. The operation for closure of the opening proved successful.

Two years later she came to me with violent cystitis, which she said began soon after the closure of the fistula. She had been treated by several physicians, medicinally, but no examination had been made. A digital examination, per vagina, detected quite a large stone in the bladder; a prolongation of the stone having been forced through the septum, and was presenting in the vagina.

I presented her the following Friday at my clinic, and by a longitudinal incision in the vesico-vaginal septum, removed a calculus measuring five and one-half inches in longest, by five inches in shortest circumference. The incision was closed, and patient made rapid and complete recovery.

A section of the stone showed most beautifully the concentric layers of mucus, from the inflamed bladder wrapped around the phosphatic nucleus, and each layer thoroughly sanded with phosphates.

CASE VII.—URETHRAL CALCULUS, URIC ACID—WEIGHT 140 GRS.

Henry Gates, colored, 47 years of age, general condition very poor. Had complained of frequent desire to pass water, with small stream, for upwards of two years. Had been examined for stricture, but physician was unable to reach the bladder with small instruments.

In March, 1880, I passed No. 16 (F.) steel sound, and detected urethral calculus in the bulbous urethra. His general condition was poor, and suffering severe, the removal of the cause was a primary indication.

Perineal section, on a grooved staff, was performed; with small pair of forceps the stone was extracted, after having been worked back, by external manipulation, into the membranous urethra. The bladder was explored; no other calculus found; the stricture was then divulsed, and No. 29 sound passed.

The patient did badly, continued slowly downward, in spite of support, and died on the eighteenth day. No further symptoms of bladder or urethral trouble, but rather a hectic fever, coming on every evening.

Was unable to obtain post mortem, but believe some renal trouble existed.

CASE VIII.—VESICAL CALCULUS, URIC ACID—WEIGHT 490 GRS.

Jas. H—, male, colored, 38 years of age, from one of the river counties of Mississippi, presented himself at my College clinic with symptoms clearly indicative of foreign body in bladder, with blood in his urine, and great pain in his loins. He suffered intensely as the last few drops of water were coming from his bladder, with a sensation of a piece of gravel at the head of the penis. These symptoms had extended over a period of three years. He had been treated by several physicians, but no examination of the bladder had been made. He was examined before the class, and the presence of stone was confirmed by the click of the steel sound. His general condition was fair, but the operation was postponed for a day or two, in order that he should be thoroughly quininized, as he was from a malarial district. He was presented at my next clinic. The median incision was made in the bladder. The stone was found quite large, and in order to remove it without too much lasceration of the prostate gland, I incised left side, and succeeded in delivering the stone.

The hemorrage amounted to almost nothing; nor was there any schock following the operation. The following day he had a rigor, which was followed by a temperature of 105, but which yielded to antifebrin. He was kept under the influence of opium and quinine for three or four days. At the end of the second week he was presented to the class; the wound having almost healed, he left for his home.

Two months later he returned with a small fistulous opening in perineum, through which a few drops of water escaped while urinating. I introduced a thirty-two steel sound to the bladder once, and the third day the fistnla had healed, and the patient returned home. The calculus was egg-shaped, measuring — by — inches in both short and long circumferences.

CASE IX.—VESICAL CALCULUS; PHOSPHATIC.—WT. 550 GRS.

Henry Green, male; colored; thirteen years; was sent to my college clinic by Dr. Watson, of Miss. It seems that his bladder trouble had existed for five or six years. Dr. Watson was the first to diagnose the stone, which was confirmed, and the doctor having had him on a preparatory treatment for some days, he was at once anæsthetised, and the lateral operation was performed. Nothing worthy of mention, save rapid recovery, occurred in this case. The patient returned home on the eight day. The stone measured four by six inches in its short and long circumferences. I learned afterwards that a small perineal fistula remained, but could never have him brought back for the introduction of instruments. The boy's health being perfect, he did not seem to attach much importance to a few drops of urine on his clothes.

CASE X.—VESICAL CALCULUS; PHOSPHATIC.—WT. — GRS.

Mrs. M—, white; fifty-eight years; was called to examine her in 1882, by her physician.

In 1869 she was cured of a vesico-vaginal fistula, of twenty years standing,—operation by my father; since which time her health has been good, and only within the year of my examination had she suffered with cystitis.

The searcher was passed to the bladder and calculus detected. Under anæsthesia the urethra was dilated and calculus extracted.

No dribbling of urine followed, and she was up and entirely well in two days.

CASE XI.—URETHRAL CALCULUS; LITHIC ACID.—WT. 110 GRS.

Sidney F—, male; white; aged six years; residence, city; was brought to me in July, 1878. For four years he had complained of great pain at the close of each of very frequent acts of micturition, and for six weeks had experienced great difficulty in passing his water. Examining his penis with a suspicion of adherent prepuce, I detected an enlargement in the body of the organ, about midway between the meatus and scrotum, and which, upon my passing a probe down the urethra, proved to be a stone. I endeavored, by manipulation, to work the stone toward the meatus, but failed. It could be pressed backward, toward, and presumably into the bladder. I believed the forcible dilitation and extraction, by means of forceps, would damage the canal more than a simple incision; hence, the following day performed external urethrotomy at the point farthest forward to which the stone could be pressed. The incision was longitudinal and was closed with silver wire. No urine passed through the opening, and primary union took place.

The stone measured 1⅜ by 1⅛ inches—its two circumferences. Quite large for urethra of six year old boy.

CASE XII.—VESICAL CALCULUS; PHOSPHATIC.—WT. 270 GRS.

Edward Mc—, M.; white; twenty-six years; entered my private infirmary upon the advice of Dr. Gladden, of Tipton county, Tenn., February 26; 188–.

The patient was very much emaciated, suffering with most violent cystitis, which had begun in boyhood. The diagnosis of stone was readily confirmed with the introduction of sound; but his condition was such that we delayed the operation some days, in order to build him up. He improved very slowly, if at all, and as the removal of stone held out the only hope of checking a fast-wasting life, an operation was advised and agreed to.

The lateral operation was performed, the stone was crushed, and all fragments removed. The pain and bladder trouble ceased and he rallied. His condition was quite hopeful for six or seven days, when diarrhœa came on, followed by dysentery, and hectic fevers, with sweats and rigors. In spite of stimulants and nourishments, the patient gradually faded away, dying on the 24th day after the operation. No post-mortem was obtained. The fragments of stone weighed 270 grains.

This was a case in which, without doubt, an early operation would have saved the patient's life.

CASE XIII.—VESICAL CALCULUS, LITHIC ACID—WEIGHT 260 GRS.

J. W—, male, white, 4 years of age, was brought to me by Dr. Johnson, of Arkansas. For two years the patient had presented prominently the usual symptoms of vesical calculus. Repeated examinations had been made, and different diagnosis given as to the presence of stone. Patient's general health, excellent. With a pocket probe for a sound, I detected a stone at once, on entering his bladder; and four days later I presented the little fellow at my college clinic, at the Memphis Hospital Medical College. The bladder was injected with four ounces of warm borated water, a small staff introduced, and on it the median lithotomy performed. On grasping the stone, I found it too large for safe abstraction through the median incision alone. With a blunt pointed bistoury, guided by my left index finger, I incised the neck of the bladder—the miniature prostate—bilaterally. The stone was then grasped with forceps, and abstracted. The bladder was irrigated with hot bi-chloride solution. The hemorrhage was very slight; shock, *nil.* The first evening his temperature stood 100½; the morning following, his temperature was normal. In four days he was up, and on the eighth day, the wound was granulating nicely, he was taken home. The flow of urine was under control. The stone was oval, and rather smooth; measured 7/8x1 1/8x1 5/8 inches in its various diameters. Two month later the patient was entirely well.

CASE XIV.—VESICAL CALCULUS, URIC ACID—WEIGHT 520 GRS.

Chas. J—, male, white, 58 years of age, resident of this city, a man of active temperament and unusual sound physique. I was consulted by him in 1883, for cystitis, which had been gradually growing worse for about four years. A diagnosis of stone was made with sound, and after some days of preparatory treatment, without an anæsthetic, I attempted to fill his bladder with warm carbolized solution, but found that even so small a quantity as four ounce gave him most intense pain. This at once discarded the idea of supra-pubic cystotomy. A few days later he was anæsthetized, and lithotripsy attempted. The stone was seized with lithotrite, but I found it impossible to crush it. A grooved staff was then introduced, and the lateral operation for stone performed. Finding the stone too large for removal, I incised the prostate gland on the other side, and then succeeded, after considerable traction, in removing the stone.

The venous hemorrhage was considerable. By means of a skirted catheter, the tampon was used. He rallied, and did well for the first ten days, and was able to sit up; but from that time on he gradually grew worse. Rigors came on, followed by fevers and sweats, and the usual train of hectic symptoms. He died on the thirty-eighth day after the operation. No post-mortem.

CASE XV.—VESICAL CALCULUS; PHOSPHATIC.—WT. 430 GRS.

F. Hollnigs, male; white; aged 15; for some years a sufferer with cystitis. In October last he came under the care of Dr. Smith, of Arkansas City, Ark., who diagnosed calculus as the cause of cystitis and sent him to my infirmary for operation. Six days were given to preparatory treatment, when, on November 18, I

operated per perineum, removing by the median incision a phosphatic stone, weighing 430 grains. His recovery was not rapid, but he went home at the end of four weeks quite well.

